# Northern blotting analysis of microRNAs, their precursors and RNA interference triggers

**DOI:** 10.1186/1471-2199-12-14

**Published:** 2011-04-11

**Authors:** Edyta Koscianska, Julia Starega-Roslan, Lukasz J Sznajder, Marta Olejniczak, Paulina Galka-Marciniak, Wlodzimierz J Krzyzosiak

**Affiliations:** 1 Laboratory of Cancer Genetics, Institute of Bioorganic Chemistry, Polish Academy of Sciences, Noskowskiego 12/14 Str., 61-704 Poznan, Poland

## Abstract

**Background:**

Numerous microRNAs (miRNAs) have heterogeneous ends resulting from imprecise cleavages by processing nucleases and from various non-templated nucleotide additions. The scale of miRNA end-heterogeneity is best shown by deep sequencing data revealing not only the major miRNA variants but also those that occur in only minute amounts and are unlikely to be of functional importance. All RNA interference (RNAi) technology reagents that are expressed and processed in cells are also exposed to the same machinery generating end-heterogeneity of the released short interfering RNAs (siRNAs) or miRNA mimetics.

**Results:**

In this study we have analyzed endogenous and exogenous RNAs in the range of 20-70 nt by high-resolution northern blotting. We have validated the results obtained with northern blotting by comparing them with data derived from miRNA deep sequencing; therefore we have demonstrated the usefulness of the northern blotting technique in the investigation of miRNA biogenesis, as well as in the characterization of RNAi technology reagents.

**Conclusions:**

The conventional northern blotting enhanced to high resolution may be a useful adjunct to other miRNA discovery, detection and characterization methods. It provides quantitative data on distribution of major length variants of abundant endogenous miRNAs, as well as on length heterogeneity of RNAi technology reagents expressed in cells.

## Background

MicroRNAs (miRNAs) are endogenous short RNAs (~22 nt) that control gene expression at the posttranscriptional level. There is growing evidence that miRNAs regulate various physiological processes and are frequently misregulated in many diseases [1-9]. The biogenesis of animal miRNAs includes two RNA cleavage steps (reviewed in [10-13]). First, in the nucleus, primary miRNA transcripts (pri-miRNA) are cleaved into approximately 60 nucleotide-long pre-miRNA precursors by the ribonuclease Drosha acting together with DGCR8 protein within the complex named Microprocessor [14,15]. Then, the pre-miRNAs are exported to the cytoplasm by Exportin-5 [16,17] and cleaved further by the ribonuclease Dicer protein complex into ~20 nucleotide-long miRNA duplexes [18,19]. One of the two RNA strands becomes functional miRNA via Argonaute protein binding, and the other is released and degraded [20,21]. Mature miRNAs are heterogeneous in length, varying between 19 and 25 nt [22-25]. The primary source of miRNA length heterogeneity is imprecise cleavage by the ribonucleases Drosha and Dicer [26]. Further, miRNA 5'-end selection occurs upon Argonaute protein binding [27]. The miRNAs that differ in their 5'-ends have different seed sequences and may regulate different sets of targets [24,28-30]. Detection of the cellular levels of individual length variants of miRNAs with high precision is therefore very important. Similarly, determination of the exact length distribution of reagents released from the vectors used in RNAi and miRNA technologies is of importance because it may influence their performance in cells [31]. It is also advantageous to monitor the lengths of reagents released from the vectors with regard to the off-target effects that these products may cause [32,33].

Numerous reports have described various improvements of the northern blotting technique [34-39]. In this study, we use the method refined for extremely high-resolution detection of miRNAs, pre-miRNAs, siRNAs released from vectors, and any short RNAs of corresponding lengths. We demonstrate the usefulness of this northern blotting procedure by showing examples of its application in miRNA and RNAi fields to evaluate the precision of Drosha and Dicer cleavages.

## Results and discussion

We show here that northern blotting of short RNAs that are 20-70 nt in length may provide insightful information on the distribution of individual length variants of siRNAs, miRNAs and their precursors in cells. We first show that high-resolution northern blotting and deep sequencing give similar results for abundant miRNAs. Then, we advance our recent observations showing utility of this northern blotting protocol for evaluating precision of Drosha and Dicer cleavages during human miRNA biogenesis [26]. Finally, we put special emphasis on the need for better characterization of reagents released from expression constructs used to activate RNAi in cells.

### Correlation between high-resolution northern blotting and deep sequencing results

**Figure 1:**
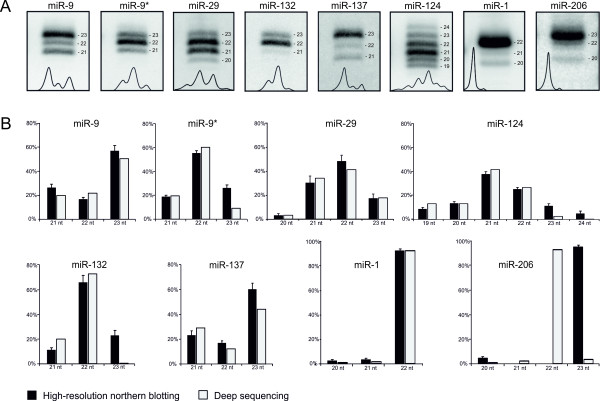
**Correlation between high-resolution northern blotting and deep sequencing results**. A) Appropriately cropped representative northern pictures for endogenous mouse neuromiRs: miR-9, miR-9*, miR-29, miR-124, miR-132, miR-137, and myomiRs: miR-1, and miR-206 are shown. B) Comparative analysis of the relative distribution of miRNA length variants. The percentage of the various length variants of mouse miR-9, miR-9*, miR-29, miR-124, miR-132, miR-137, miR-1, and miR-206 observed in our high-resolution northern blot detections are shown as black bars; equivalent miRNAs identified by deep sequencing are shown as gray bars. Relative shares of miRNA length variants (denoted as nt) are calculated in percentage (%). Standard errors are from twelve independent northern blot samples for miR-9, miR-9*, miR-29, miR-124, miR-132, miR-137, from nine samples for miR-1, and from three samples for miR-206. One-nucleotide discrepancy between northern blotting and deep sequencing results observed in the case of miR-206 may be due to a difference in the migration rate of this miRNA, which probably results from its nucleotide composition.

An increasingly popular high-throughput technology for miRNA discovery and expression profiling is deep sequencing [22-25]. To validate high-resolution northern blotting as a suitable method for miRNA length heterogeneity studies, we compared the results obtained using this method with the deep sequencing results obtained by others using Illumina sequencing-by-synthesis platform for miRNA discovery in mice [24]. The following endogenous mouse miRNAs that differ in length heterogeneity have been analyzed: miR-9, miR-9*, miR-29, miR-124, miR-132 and miR-137 specific for neuronal tissues, as well as miR-1 and miR-206 specific for muscle tissues (Figure 1). Total RNAs were extracted from selected brain sections (cortex, cerebellum, striatum and thalamus) or muscle tissues (heart and skeletal muscles from legs), and the miRNAs abundantly expressed in these tissues were detected by northern blotting with specific probes. To evaluate relative levels of miRNA heterogeneity, radioactive northern blotting signals were quantified by phosphorimaging and the percentages of individual length variants in the miRNA fractions were calculated. Similarly, the distribution of miRNA length variants was calculated from deep sequencing data [24] (Additional file 1: Supplemental Table S1). These two datasets correlated well with each other (Figure 1). However, it should be borne in mind that the degree of correlation may vary when a different sequencing platform and/or data filtering system is used. Of all the analyzed miRNAs, length heterogeneity was greatest for miR-124, which is in accordance with deep sequencing results showing that some miRNAs have multiple isoforms [22,24,25]. In contrast to deep sequencing, northern blotting shows only the most abundant miRNA variants. This feature may be considered advantageous because only highly expressed miRNA variants are of functional importance [24].

### Application of northern blotting in studies of miRNA and pre-miRNA length heterogeneity

**Figure 2:**
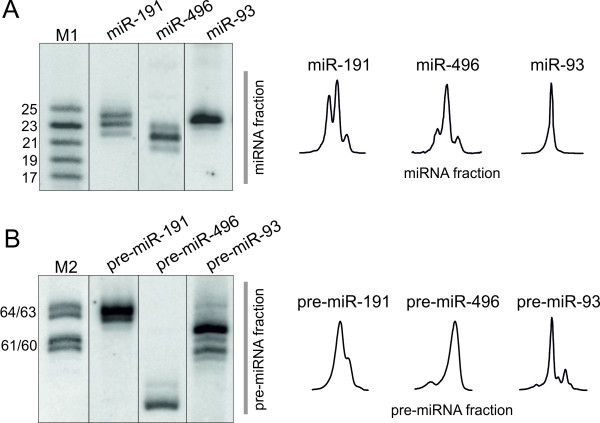
**Detection of heterogeneous miRNAs and pre-miRNAs**. Examples of heterogeneous miRNAs and pre-miRNAs overexpressed in HEK 293T cells and detected with single-nucleotide resolution, as shown in the figure. A) miRNAs: miR-191, miR-496, miR-93 resolved in the range of ~17-25 nt. B) pre-miRNAs: pre-miR-191, pre-miR-496, and pre-miR-93 resolved in the range of ~60-70 nt. M-lanes denote the appropriate radiolabeled RNA oligonucleotide markers (ORNs); M1 denotes 17, 19, 21, 23, 25-nt ORNs for miRNA detection (A) and M2 denotes 60/61 and 63/64-nt ORNs for pre-miRNA detection (B). Quantitative representations of miRNA and pre-miRNA variants obtained from phosphorimaging analyses are shown schematically.

Northern blotting analysis with single-nucleotide resolution makes it possible to detect both miRNA and pre-miRNA length variants. We have therefore found this method useful in our studies of molecular sources of miRNA length heterogeneity [26]. Such heterogeneity has already been observed by deep sequencing in the case of miRNAs [23,29] and by cloning in the case of pre-miRNAs [40]. The pre-miRNA heterogeneity has its primary source in the imprecise pri-miRNA cleavage by Drosha [41,42]. The miRNA heterogeneity results from imprecise cleavages by both Drosha and Dicer and can be further biased at the AGO2 programming step [27] and by various post-cleavage modifications, such as an untemplated nucleotide addition [22,43]. Here, we show examples of northern blots of endogenous mouse miRNAs (Figure 1A), as well as of miRNAs and pre-miRNAs overexpressed from vector constructs (Figure 2). We used the miRNA overexpression system because only a limited number of known miRNAs are expressed in a given cell line or tissue, and only a fraction of these miRNAs are expressed at levels detectable by northern blotting. Hence, the use of a miRNA overexpression system may assist miRNA biogenesis studies by increasing cellular levels of miRNAs and, more importantly, facilitating the detection of miRNA precursors. The miRNAs and pre-miRNAs, shown here as examples, reveal different levels of length heterogeneity. Specifically, three length variants were observed for miR-191 and miR-496, whereas only one was seen for miR-93 (Figure 2A). Similarly, individual pre-miRNAs differed in the number of length variants (Figure 2B). The level of length heterogeneity observed here is in agreement with the results of our large-scale analysis of Drosha and Dicer cleavage specificities [26]. The length heterogeneity detected by northern blotting can be quantitatively evaluated by analyzing appropriate signal intensities (Figure 2, right panels), making high-resolution northern blotting an even more reliable method.

### High-resolution northern blotting in siRNA studies

**Figure 3:**
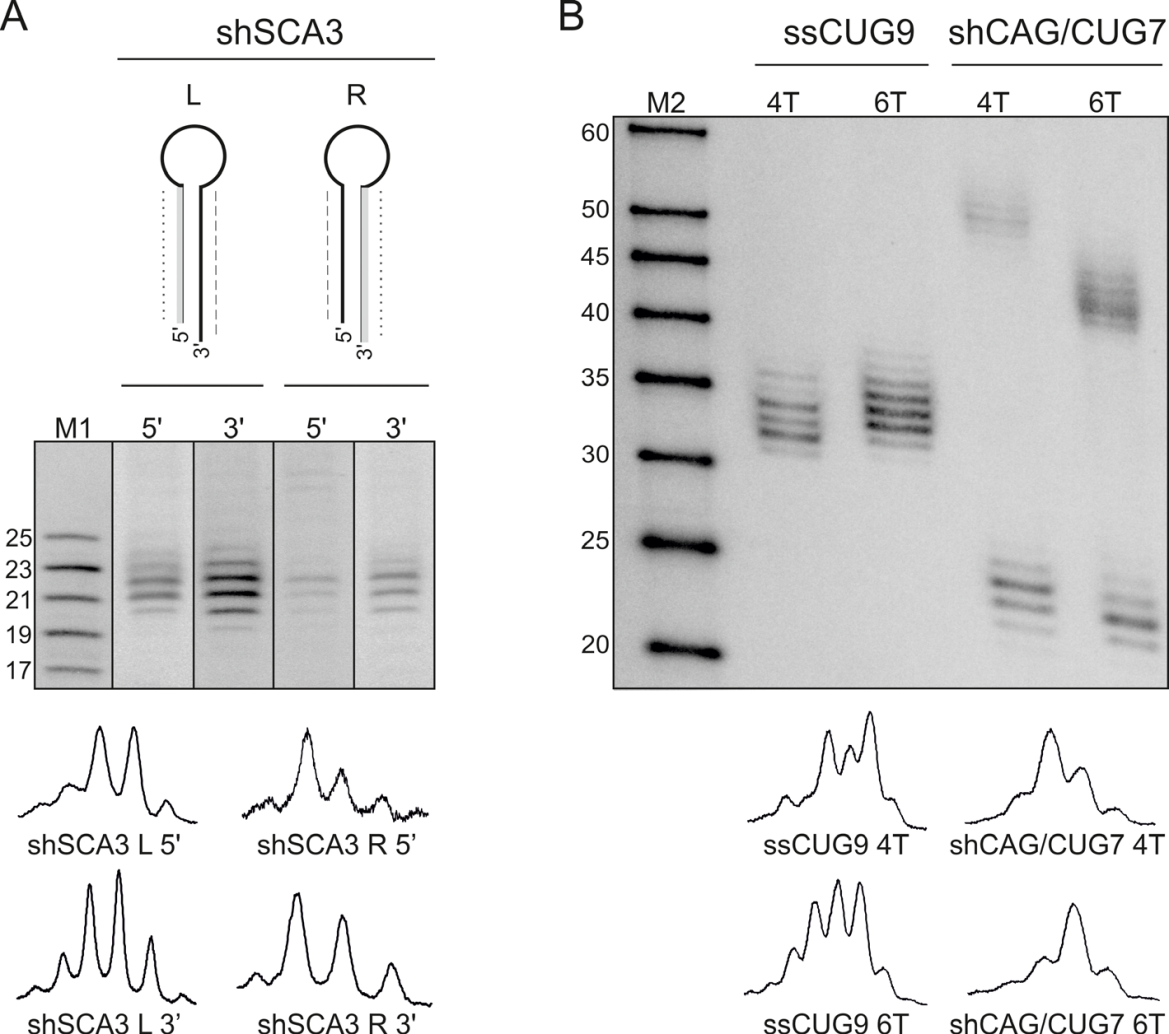
**Application of the high-resolution northern blotting in monitoring RNA reagents expressed in cells**. A) Processing of antisense-sense and sense-antisense shRNAs (L-shSCA3 and R-shSCA3, respectively). The 5' and 3' strands of each shRNA were analyzed by detection with probes complementary to either siRNA strand, as indicated in the figure. One probe detected L-5' and R-3' strands (dotted line) while the other probe detected L-3' and R-5' strands (dashed line). B) ssRNA CUG9 and shRNA CAG/CUG7 transcribed from vectors, having either 4T or 6T at their termination sites. M denotes size markers; M1 denotes end-labeled 17, 19, 21, 23, and 25-nt synthetic RNA oligonucleotides and M2 denotes RNA Low Molecular Weight Marker (USB). Quantitative representations of siRNA length variants are shown in the bottom panel, using peaks obtained from phosphorimaging analyses.

Short hairpin RNAs (shRNAs) may be constructed in two ways; they may have antisense (guide) strands in the 3' or 5' arm of the shRNA stem (discussed in [44]). We designed both sense-antisense (R-type) and antisense-sense (L-type) shRNAs having the same guide sequence specific for spinocerebellar ataxia type 3 (ATXN3) mRNA (for simplicity, these shRNAs are hereinafter referred to as R-shSCA3 and L-shSCA3) and cloned them into a p*Silencer *vector (Ambion). To estimate the length of siRNAs excised from the shRNAs by Dicer, we transfected HEK 293T cells with the vector constructs and performed high-resolution northern blotting analysis. Probes detecting either the sense or antisense strand of R-shSCA3 and L-shSCA3 were used for northern blotting (Figure 3A). The siRNAs generated from both types of shRNAs were heterogeneous in length, and their length variants were easily distinguishable at 1-nt resolution. The hybridization signals obtained by detection with appropriate probes were unequal in intensity, but the distribution of particular length variants was only slightly different, regardless of the location of the shRNA guide strand. It should be borne in mind that the observed siRNA length heterogeneity may result not only from imprecise Dicer cleavage of the shRNA but also from heterogeneity at its 3'end to which the PAZ domain of Dicer anchors. Therefore, the monitoring of RNA reagents released from shRNAs is important. The shRNAs encoded by appropriate vectors are transcribed from U6 or H1 RNA polymerase III (Pol III) promoters [45]. The start of transcription is strictly defined as the +1 position of the promoter in the vector, but termination is less accurate and occurs within a short stretch of several uracil residues in the transcript [46]. Hence, the length heterogeneity of shRNAs may be solely the result of the different lengths of the oligo-U tails at the 3' end. Using high-resolution northern blotting, we analyzed shRNAs and their siRNA products generated from vectors with transcription termination signals composed of different numbers of thymidines (4T and 6T). We transfected HEK 293T cells with vectors carrying either single stranded RNA composed of nine CUG repeats (ssRNA CUG9) or double stranded RNA forming a hairpin, composed of seven CAG/CUG repeats (shRNA CAG/CUG7). These constructs were driven by the H1 promoter but with either 4T or 6T stretches as the termination signal (Figure 3B). As we expected, the ssRNA CUG9 transcript not forming a hairpin with a distinctive loop was not processed by Dicer, and we observed heterogeneous products with a length range of ~30-35 nt that were derived solely from unspecific transcription termination. We also observed length heterogeneity in siRNA products released from both shRNA constructs. Moreover, the pattern of length distribution varied. In the case of the shCAG/CUG terminating at 4T, the longer siRNA variants (~23-24 nt) dominated in the siRNA pool, whereas in the case of the same shRNA terminating at 6T, shorter siRNA variants (~22-23 nt) were more abundant. These examples show that several obstacles to achieving effective shRNA design still need to be overcome [47,48], and high-resolution northern blotting may be helpful in addressing these issues. The imprecise processing of shRNAs in cells has important implications for siRNA technology because it results in the production of only a fraction of silencing reagent with the desired sequence. This issue is especially important for allele-specific applications of RNAi technology in which a transcript is targeted at a single-nucleotide polymorphism (SNP) linked to a mutation.

## Conclusions

In this study we have shown that the optimized high-resolution northern blotting can be used to analyze endogenous and exogenous RNAs in the range of 20-70 nt. We demonstrated the usefulness of this technique in the investigation of miRNA biogenesis as well as in the characterization of RNAi technology reagents. We have validated the high-resolution northern blotting as a reliable tool for length heterogeneity analysis of miRNAs and their precursors and have presented examples of its application in miRNA and siRNA studies. The method can be used in a variety of applications to verify mechanisms of RNAi-mediated effects. However, we understand that there is a limit to the interpretation of northern blots, and other techniques have to be used to provide complementing information. The techniques allowing precise mapping of both 5' and 3' ends of processed products include deep sequencing, primer extension (5') and rapid amplification of cDNA ends (5' and 3' RACE). These methods, when used jointly, will provide more complete and more reliable information about the exact lengths and end-sequences of miRNA and cell-expressed siRNA variants. Such information is very important as miRNA and siRNA variants having different 5' ends may differ in the potency to activate RISC [27,49], as well as in downstream silencing effects. RISC programmed by different miRNA 5'-end variants may regulate different targets [24,28-30], and programmed by siRNA variants may cleave mRNAs at shifted sites compromising the allele-specific SNP-targeting applications.

## Methods

### Animals

The animals were kept under standard conditions with a 12-h light/dark cycle and water and food ad libitum. The animals were sacrificed by placing them in a 70% CO_2 _atmosphere. The original strains C57BL/6J and C3H/HeJ were obtained from The Jackson Laboratory (Bar Harbor, Maine; USA) and were bred to B6C3F1.

The study was carried out in strict accordance with Polish Law on Animal Experimentation which complies with EU standards. All procedures and animal handling were carried out to minimize animal stress and were approved and monitored by The Local Ethical Commission for Animal Experiments in Poznan (Decision Number: 49/2010).

### Cell culture

HEK 293T cells were obtained from the American Type Culture Collection (ATCC) and grown in Dulbecco's Modified Eagle's Medium (DMEM, Lonza) with 10% fetal bovine serum (FBS, Sigma-Aldrich) and Antibiotic Antimycotic Solution (Sigma-Aldrich) at 37°C in a humidified atmosphere of 5% CO_2_.

### DNA transfection

HEK 293T cells were grown to 90% confluence in T-25 flasks and transfected with 3 μg of either plasmid constructs (System Biosciences) encoding appropriate miRNA precursors or plasmid vectors (p*Silencer *3.1-H.1 hygro, Ambion) containing specific expression cassettes (shRNA) (Additional file 1: Supplemental Table S2), using Lipofectamine 2000 (Invitrogen). The cells were harvested 24 hours after transfection, and isolated RNAs were analyzed by northern blotting.

### RNA isolation and northern blotting of miRNAs and pre-miRNAs

Total RNA was extracted from the cells and selected mouse brain and muscle tissues using TRI Reagent (MRC, Inc., BioShop) according to the manufacturer's instructions. RNAs (20-30 μg) were resolved on denaturing polyacrylamide gels (12% PAA, 19:1 acrylamide/bis, 7 M urea) in 0.5 × TBE. Two separate electrophoresis runs were performed, as described previously [26,50]. Briefly, a vertical electrophoresis gel system (II xi Cell, BioRad) for resolution of miRNAs, and a model S2 sequencing gel electrophoresis apparatus (Gibco, Life Technologies) for pre-miRNA separations were used. Xylene cyanol dye (XC) migrated 10 cm and 30 cm, for high resolution of miRNA and pre-miRNA fractions, respectively. Marker lanes contained a mixture of simultaneously radiolabeled synthetic RNA oligonucleotides (ORNs: 17-, 19-, 21-, 23-, 25-nt or ORNs: 60-, 61-, 63-, 64-nt) or RNA Low Molecular Weight Marker (USB). RNAs were transferred to GeneScreen Plus hybridization membrane (PerkinElmer) using semi-dry electroblotting (Sigma-Aldrich), immobilized by subsequent UV irradiation (120 mJ/cm^2^) (UVP) and baked in an oven at 80°C for 30 min. The membranes were probed with specific oligodeoxynucleotides (ODNs) complementary to the annotated mouse miRNAs miR-9, -9*, -93, -29a, -29b, -124, -132, -137, -191, -496, -1 and -206 (miRBase) and to 21-nt siRNAs generated from shRNAs (Additional file 1: Supplemental Table S3). The ODNs were labeled with [γ^32^P] ATP (5000 Ci/mmol, Hartmann Analytics) using OptiKinase (USB) according to the manufacturer's instructions. Pre-hybridizations and hybridizations were carried out under the same conditions at 37°C overnight in buffer containing 5 × SSC, 1% SDS and 1 × Denhardt's solution. After hybridization, the membranes were washed three times in a low-stringency buffer solution (2 × SSC and 0.1% SDS) for 20 minutes. Radioactive signals were quantified by phosphorimaging (Multi Gauge v3.0, Fujifilm).

## Authors' contributions

EK, JS-R, LJS, MO, PG-M performed experiments and analyzed data. EK drafted the manuscript. WJK conceived the study, supervised the research and contributed in the preparation of the paper. All authors read and approved the final manuscript.

## Supplementary Material

Additional file 1 **Supplemental tables S1, S2 and S3**. Click here for file
