# Supplementary material for: Northern blotting analysis of microRNAs, their precursors and RNA interference triggers
Source: BMC Mol Biol. 2011 Apr 11;12:14. doi: 10.1186/1471-2199-12-14 (PMC3080303; doi:10.1186/1471-2199-12-14)
Supplement: Additional file 1 — Supplemental tables S1, S2 and S3. [file 1471-2199-12-14-S1.DOC]

**Supplemental tables**

**Table S1. The most abundant sequences of analyzed miRNAs**

| **miRNA** | **Length** | **Sequence** | **Relative shares of sequence lengths (%)** | |
| --- | --- | --- | --- | --- |
|  |  |  | **deep sequencing*** | **northern blotting** |
| miR-1 | 20 nt | UGGAAUGUAAAGAAGUAUGU | 0,83 | 2,86 |
|  | 21 nt | UGGAAUGUAAAGAAGUAUGUA | 1,40 | 3,83 |
|  | 22 nt | **UGGAAUGUAAAGAAGUAUGUAU** | 92,74 | 93,32 |
| miR-9 | 21 nt | UCUUUGGUUAUCUAGCUGUAU | 19,14 | 26,44 |
|  | 22 nt | UCUUUGGUUAUCUAGCUGUAUG | 19,10 | 16,58 |
|  |  | CUUUGGUUAUCUAGCUGUAUGA | 2,67 |  |
|  | 23 nt | **UCUUUGGUUAUCUAGCUGUAUGA** | 50,43 | 56,98 |
| miR-9* | 21 nt | UAAAGCUAGAUAACCGAAAGU | 16,72 | 18,81 |
|  |  | AUAAAGCUAGAUAACCGAAAG | 2,38 |  |
|  | 22 nt | **AUAAAGCUAGAUAACCGAAAGU** | 32,39 | 55,14 |
|  |  | UAAAGCUAGAUAACCGAAAGUA | 27,80 |  |
|  | 23 nt | AUAAAGCUAGAUAACCGAAAGUA | 6,01 | 26,05 |
|  |  | UAAAGCUAGAUAACCGAAAGUAA | 2,78 |  |
| miR-29 | 20 nt | UAGCACCAUCUGAAAUCGGU | 2,10 | 3,38 |
|  | 21 nt | UAGCACCAUCUGAAAUCGGUU | 29,91 | 30,51 |
|  |  | UAGCACCAUUUGAAAUCGGUU | 2,67 |  |
|  | 22 nt | **UAGCACCAUCUGAAAUCGGUUA** | 27,21 | 48,54 |
|  |  | UAGCACCAUUUGAAAUCAGUGU | 8,63 |  |
|  |  | CUAGCACCAUCUGAAAUCGGUU | 3,93 |  |
|  |  | **UAGCACCAUUUGAAAUCGGUUA** | 1,13 |  |
|  | 23 nt | **UAGCACCAUUUGAAAUCAGUGUU** | 14,31 | 17,57 |
|  |  | UAGCACCAUCUGAAAUCGGUUAU | 1,65 |  |
|  |  | CUAGCACCAUCUGAAAUCGGUUA | 1,15 |  |
| miR-124 | 19 nt | UAAGGCACGCGGUGAAUGC | 12,30 | 8,62 |
|  | 20 nt | **UAAGGCACGCGGUGAAUGCC** | 7,58 | 13,33 |
|  |  | UUAAGGCACGCGGUGAAUGC | 4,28 |  |
|  | 21 nt | UAAGGCACGCGGUGAAUGCCA | 35,92 | 37,14 |
|  |  | UUAAGGCACGCGGUGAAUGCC | 4,07 |  |
|  | 22 nt | UAAGGCACGCGGUGAAUGCCAA | 16,65 | 24,88 |
|  |  | UUAAGGCACGCGGUGAAUGCCA | 9,59 |  |
|  | 23 nt | UUAAGGCACGCGGUGAAUGCCAA | 2,01 | 11,07 |
|  | 24 nt | UAAGGCACGCGGUGAAUGCCAAGA | 0,29 | 4,97 |
| miR-132 | 21 nt | UAACAGUCUACAGCCAUGGUC | 16,27 | 11,16 |
|  |  | AACAGUCUACAGCCAUGGUCG | 3,63 |  |
|  | 22 nt | **UAACAGUCUACAGCCAUGGUCG** | 72,64 | 65,89 |
|  | 23 nt | UAACAGUCUACAGCCAUGGUCGC | 0,49 | 22,95 |
| miR-137 | 21 nt | UUAUUGCUUAAGAAUACGCGU | 27,22 | 23,06 |
|  |  | AUUGCUUAAGAAUACGCGUAG | 1,09 |  |
|  | 22 nt | UUAUUGCUUAAGAAUACGCGUA | 7,70 | 16,83 |
|  |  | UAUUGCUUAAGAAUACGCGUAG | 4,19 |  |
|  | 23 nt | **UUAUUGCUUAAGAAUACGCGUAG** | 39,82 | 60,11 |
|  |  | UAUUGCUUAAGAAUACGCGUAGU | 4,09 |  |
| miR-206 | 20 nt | GAAUGUAAGGAAGUGUGUGG | 0,56 | 4,42 |
|  | 21 nt | UGGAAUGUAAGGAAGUGUGUG | 2,18 | n/d |
|  | 22 nt | **UGGAAUGUAAGGAAGUGUGUGG** | 93,41 | n/d |
|  | 23 nt | AUGGAAUGUAAGGAAGUGUGUGG | 2,03 | 95,58 |
|  |  | UGGAAUGUAAGGAAGUGUGUGGU | 1,37 |  |

The distribution of miRNA length variants calculated in percentage (%) on the basis of our high-resoulution northern blotting results and deep sequencing data (Chian*g et* al., 2010). The miRNA sequences annotated in miRBase are bolded.

* Chiang HR, Schoenfeld LW, Ruby JG, Auyeung VC, Spies N, Baek D, Johnston WK, Russ C, Luo S, Babiarz JE, Blelloch R, Schroth GP, Nusbaum C, Bartel DP. 2010. Mammalian microRNAs: experimental evaluation of novel and previously annotated genes. *Genes Dev*. 24(10):992-1009

**Table S2. Sequences of shRNAs and ssRNAs carried by plasmid vectors**

| **shRNA/ssRNA** | **Cloned sequence** |
| --- | --- |
| shSCA3L | AUAGGUCCCGCUGCUGCUGCU**CUUCCUGUCA**AGCAGCAGCAGCGGGACCUAUUU |
| shSCA3R | GCAGCAGCAGCGGGACCUAU**CUUCCUGUCA**AUAGGUCCCGCUGCUGCUGCUU |
| ssCUG9 | GCUGCUGCUGCUGCUGCUGCUGCUGCUU |
| shCAG/CUG7 | GCAGCAGCAGCAGCAGCAGCAG**CUUCCUGUCA**CUGCUGCUGCUGCUGCUGCUGCUU |

*The guide strand sequences are underlined and hairpin loop sequences are bolded

Table S3. Oligodeoxynucleotide sequences used as northern blot probes

| **Detected sequence** | **Probe sequence** |
| --- | --- |
| miR-1 | ATACATACTTCTTTACATTCCA |
| miR-9 | TCATACAGCTAGATAACCAAAGA ACTTTCGGTTATCTAGCTTTAT |
| miR-9* | ACTTTCGGTTATCTAGCTTTAT CATACAGCTAGATAACCAAAGA |
| miR-29a | TAACCGATTTCAGATGGTGCTA |
| miR-29b | AACACTGATTTCAAATGGTGCTA |
| miR-93 | CTACCTGCACGAACAGCACTTTG |
| miR-124 | GGCATTCACCGCGTGCCTTA |
| miR-132 | CGACCATGGCTGTAGACTCTTA |
| miR-137 | CTACGCGTATTCTTAAGCAATAA |
| miR-191 | CAGCTGCTTTTGGGATTCCGTTG |
| miR-206 | CCACACACTTCCTTACATTCCA |
| miR-496 | GAGATTGGCCATGTAATACTCA |
| shSCA3L 5‘ | CAGCAGCAGCGGGACCTATCT |
| shSCA3R 3‘ |
| shSCA3L 3‘ | ATAGGTCCCGCTGCTGCTGCT |
| shSCA3R 5‘ |
| ssCUG9 | AGCAGCAGCAGCAGCAGCAGC |
| shCAG/CUG7 |
